# Large-scale production of non-conventional edible plants for biodiverse school meals

**DOI:** 10.3389/fnut.2024.1282618

**Published:** 2024-04-30

**Authors:** Guilherme Reis Ranieri, Nuno Rodrigo Madeira, Betzabeth Slater, Mariana de Toledo Marchesi, Maria Angela Delgado de Oliveira, Ana Flávia Borges Badue, Thais Mauad

**Affiliations:** ^1^Department of Pathology (FMUSP), University of São Paulo, São Paulo, Brazil; ^2^Instituto Kairós, São Paulo, Brazil; ^3^EMBRAPA Vegetables, Brazilian Agricultural Research Corporation (EMBRAPA), EMBRAPA Hortaliças, Brasilia, Brazil; ^4^Department of Nutrition (FSPUSP), University of São Paulo, São Paulo, Brazil; ^5^School Feeding Department (DAN), Education Management Unit, Jundiaí, Brazil

**Keywords:** school feeding, food biodiversity, non-conventional vegetables, public policy, nutrient-rich plants, underutilized food plants, non-conventional edible plants

## Abstract

**Introduction:**

School feeding programs are important for ensuring food security and promoting child health and development, particularly in low-income countries. In view of this importance, it is possible to increase the quality of these meals by diversifying the vegetables offered and incorporating underutilized plants to improve dietary diversity and nutritional quality into school meals.

**Methods:**

This study was carried out using the action research methodology following the implementation and development of the “Inova na Horta” project in the city of Jundiaí, São Paulo, Brazil. The project was based on the existing and functioning physical and organizational structure of a municipal organic farm. Vegetables were selected from among 210 non-conventional species and varieties, which were further selected for continuous production based on 8 nutritional, culinary and cultivation criteria.

**Results:**

Thirty-four vegetables were selected for continuous cultivation and provisions to the school kitchens. Nine tons of vegetables were produced and provided to 90 municipal schools from 2020–2023. Leafy vegetables accounted for most the production, with a total weight of 6441 kg corresponding to 71.6% of the total harvest. Kitchen teams were trained throughout the project duration.

**Discussion:**

The feasibility of the production and culinary use of 34 biodiverse, nutrient-rich and underutilized food vegetables for school meals was demonstrated. The selected vegetables are nutrient-rich and contain higher amounts of minerals and proteins than the control vegetables (conventional vegetables), thus complementing several nutrients in school meals. This methodology can be replicated by municipalities of various sizes as a public policy of food and nutritional security associated with the valorization of local biodiversity.

## 1 Introduction

School feeding programs benefit children and adolescents around the world. It is estimated that several countries provide daily school meals to approximately 305 million students (1). In Brazil, the main public policy regulating school feeding is the National School Feeding Program (PNAE) (2), which aims to support child development through adequate nutrition, improving student skills and reducing dropout (3). By 2022, approximately 37 million students were provided 50 million meals daily in more than 150,000 schools, made possible by direct transfers of US$7.3 billion per year from the National Treasury (4).

School meals are an important part of the daily nutritional input for many children in Brazil. The Brazilian legislation on school meals recommends a minimum of 280 g/student/week of fresh fruits, vegetables, and greens, at least 3 days per week, as well as meals that constitute at least 30% of the daily nutritional needs for energy and macronutrients (5). Schools must spend at least 30% of their food budgets on the purchase of fresh or minimally processed foods, prioritizing purchases from small-scale or family farms.

A survey carried out in 2023 indicated that school meals were the main meal for 56% of the students in the metropolitan region of Rio de Janeiro (6). However, 79% of the students reported that schools served crackers frequently, and 36% reported that schools served ultra-processed products daily, such as artificial juices, processed sweets, sandwich cookies, crackers, and chocolate bars.

The importance of introducing biodiverse foods into school meals has been increasingly recognized (7–9). Biodiversity for food and agriculture (BFA) is indispensable to food security, sustainable development and the supply of many vital ecosystem services (10). Especially in low- and medium-income countries, the expansion of food biodiversity could help increase the supply of healthy foods in schools. The potential nutritional use of neglected and underutilized plants for improving diets, including school meals, has been previously shown in different countries, such as Brazil (11), Kenya (12) and China (13), and these plants can be exploited for their economic (income generation), ecological (lower use of inputs, more resilience) and nutritional (more nutritious) potential and could be suitable for introducing biodiversity in school meals (14–18). It is estimated that 18,956 (19) to 26,000 (20) plant species have food potential, but only 150 plant species are currently consumed on a large scale, and 80% of our food demands are met by only 12 species (21).

The literature regarding the use of the term “food biodiversity” is broad, and these species include wild food plants (22, 23), wild edible plants (24), edible weeds (25), neglected and underutilized species (26) and traditional varieties (27). In Brazil, the term non-conventional food plants or non-conventional edible plants (“plantas alimentícias não convencionais” or “PANC”) has been widely used in the literature and will be used in the present study. It encompasses cultivated and wild species, native or exotic, spontaneous or not, which can be safely used as food, including the non-conventional parts of conventional edible plants (28, 29). However, the culinary use of these plants is still incipient in Brazil.

Many vegetables currently in disuse are more nutritious than conventional vegetables (30). In addition, biodiverse and native vegetables (26) can be more resistant to adverse climatic conditions than conventional vegetables (18). These vegetables have adaptive features that promote growth under marginal conditions where most exotic vegetables fail. They have a waxy cuticle that protects against rapid moisture loss and are drought-hardy due to their excellent stomatal conductance, good recovery rate after exposure to prolonged drought, and production within a very short time after the first rains. Additionally, they require less fertilizer and pesticide than most commercial or conventional vegetables (31).

However, the national guideline “Brazilian Sociobiodiversity Native Food Species of Nutritional Value” (32) describes 82 food species, 78% of consist of native fruits (33) and lists only five leafy vegetables—Barbados gooseberry (*Pereskia aculeata*), purslane (*Portulaca oleracea*), Jewels-of-Opar (*Talinum triangulare*, *T. paniculatum*), taioba (*Xanthosoma taioba*) and jambu (*Acmella oleracea*)—all of which are easy to produce, have a short cycle, take up less space than fruit production and can be produced in school gardens.

In Brazil, despite public policies facilitating the acquisition of such foods, the purchase of biodiverse foods is still rare. Dietitians do not have knowledge on these foods or access to suppliers (34). To improve the use of these plants in school diets, it is necessary to identify species with good short- or medium-term potential for cultivation, easy forms of preparation and good nutritional composition (14–16).

Therefore, the objective of this article is to describe the “Inova na Horta” project, which developed the selection, production, and large-scale and educational use of non-conventional edible plants for school feeding on a municipal scale. We report the methodology used for vegetable selection and the feasibility of large-scale production.

## 2 Sections on policy assessment

The survey of the species used, the criteria for their selection and the quantities of vegetables supplied to the schools were based on the monitoring of the “Inova na Horta” project, as well as on data provided by the Jundiaí City Administration. This study received institutional ethical approval under n. CAAE 26388919.6.0000.0065.

The study was conducted using the action research methodology (35) during the period 2019–2023. In action research, the practice alters what is being researched while being constrained by the context of the practice (35). This methodology is characterized by constancy, and the authors of this work were also the agents of the project’s implementation and execution during its five-year duration. Action research is also characterized by an effective and immediate reaction to events as they occur (36, 37). Therefore, the criteria for the selection of species in this study were consolidated over time by the feedback provided on the cultivation conditions and harvesting and by the schools and kitchen staff.

### 2.1 Description of the study area

The study was conducted in the city of Jundiaí (São Paulo, Brazil), which is situated in the Atlantic Forest Biome within the Metropolitan Region of São Paulo. It has an estimated population of 426,935 inhabitants, with a demographic density of 858.42 people/km^2^. In 2020, the city was ranked 75th out of 5,570 Brazilian cities in terms of income. The percentage of students aged 6 to 14 years was 98.2% (38), with 50,598 students enrolled in 130 primary schools.

**Figure 1:**
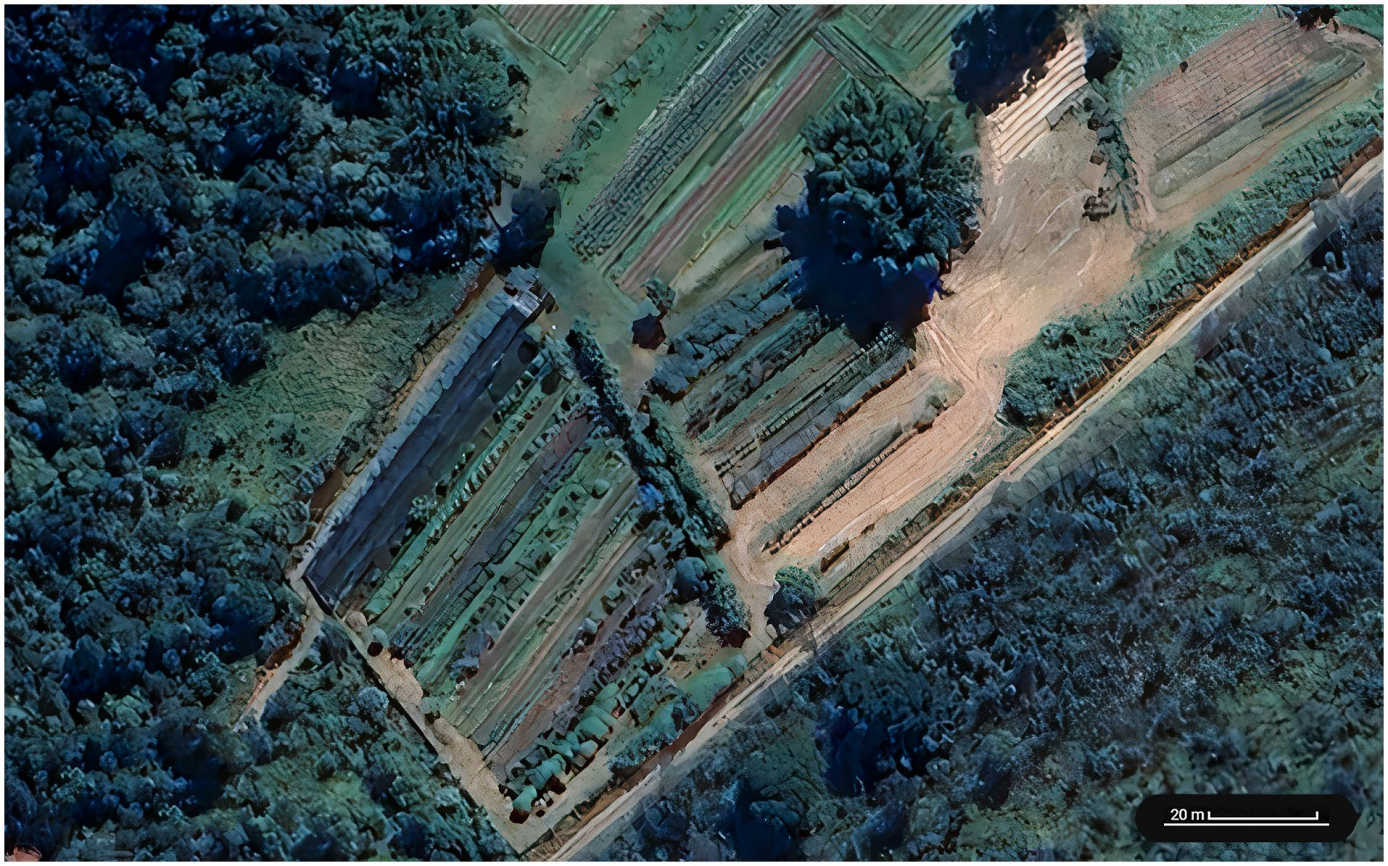
Aerial view of the Vale Verde organic farm, with delineation of the area where vegetables were grown as part of the Inova na Horta project (2019–2022). Maps Data: Google, Imagens ©2024 Airbus, CNES/Airbus, Maxar Technologies.

The production of both conventional and non-conventional vegetables to supply the local schools was carried out in Vale Verde, the Municipal Organic Farm of Jundiaí (Figure 1), whose organic production is certified by Agricontrol - OIA Brazil Certifications (Goiânia, Brazil). It is a 30,000 m^2^ site within the urban area with established vegetable delivery logistics directly to schools. This garden had an average of 20,000 m^2^ under cultivation in 2023 and represented 80% of the supply of leafy greens and vegetables for day care centers and approximately 60% for schools (Prefeitura de Jundiaí, unpublished data).

**Figure 2:**
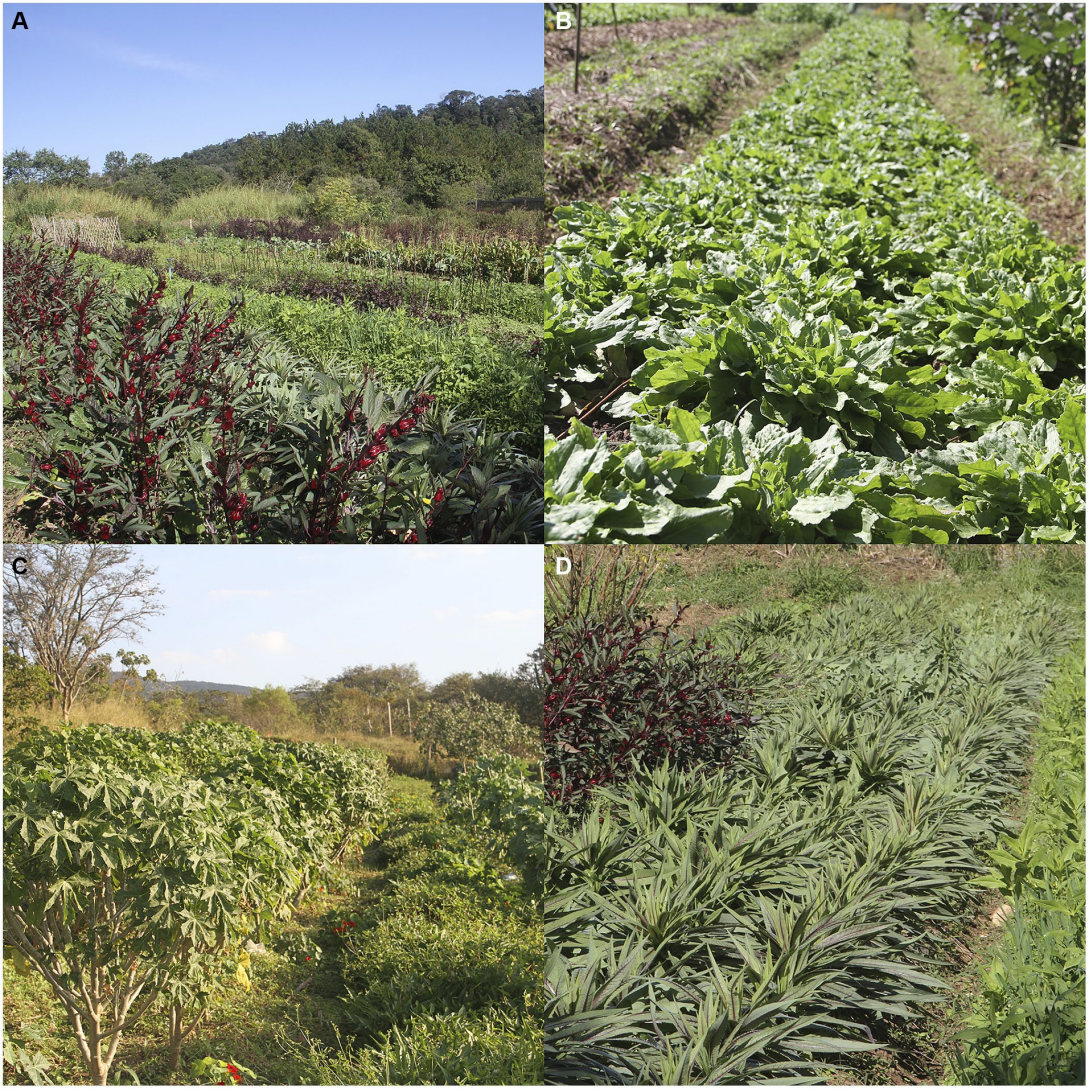
Side view of the Vale Verde municipal organic farm. The image shows the diversity of existing crops (2022), such as **(A)** *Hibiscus sabdariffa* and *Lactuca indica*; **(B)** *Rumex acetosella*; **(C)** *Cnidoscolus aconitifolius* and *Ipomea batatas*; and **(D)** *Lactuca indica*.

**Figure 3:**
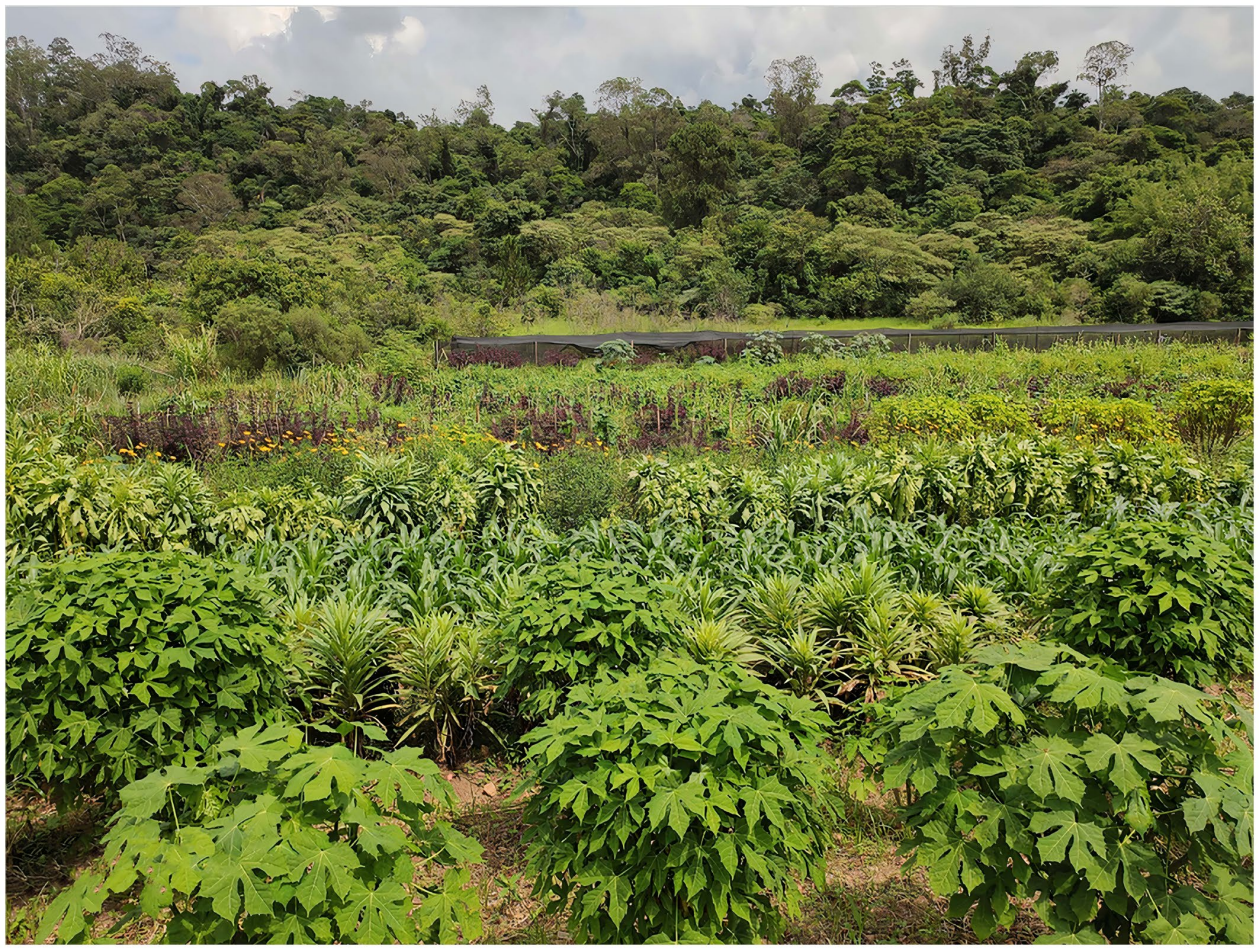
Front view of the Vale Verde municipal organic farm. This image shows the diversity of crops grown during the early summer of 2021.

The current project, “Inova na Horta,” included the production and distribution of organic cultivated non-conventional edible plants for some of the municipal schools. It was divided into crop production and training of kitchen staff and teaching staff, for the use of non-conventional edible plants in school meals (Figures 2, 3).

### 2.2 Structure

The “Inova na Horta” project was based on an existing and functioning physical and organizational structure. Production was implemented in a pre-existing field, making use of local resources such as water, irrigation systems, mechanization, tools and inputs, and electricity. It also used pre-existing distribution logistics from the field to the schools. Project costs involved setting up a plant nursery, obtaining seedlings and propagules, and costs with staff, who were responsible for coordinating field training, multiplying the plants for the school gardens, training teachers and kitchen staff, as well as administrative and accounting costs. These costs were approximately US$ 53,000/year.

### 2.3 Field methodology

Non-conventional edible plant production was carried out in a 4,000 m^2^ area starting in 2019. The production was carried out in beds that were 1 m wide × 40–60 m long. Vegetables were cultivated in a single or intercropped manner, with soil preparation, fertilization, green manure, and mulching following agroecological practices (39). The No-till system was preferred whenever possible to minimize soil disturbance and destructuring (40). In addition, green manure and mulching with shredded wood and organic plant material were used to reduce erosion and increase overall soil fertility. Crop rotation was carried out within an intercropping system, with crop rotation with green manure in each area for at least 4 months a year. Cultivation was performed by three full-time workers and two field supervisors. The workers were trained to identify, cultivate, and manage these vegetables, which require different planting and harvesting methods than conventional vegetables. We also obtained seeds and propagules from adult plants that were not commercially available, which required skilled labor in germplasm management.

### 2.4 Selection of non-conventional vegetables

Vegetable selection prioritized neglected and underutilized food plants (41) established as such based on nutrition (nutritional value and health benefits); socioeconomic (cultural acceptance and consumer preferences, access to markets and potential income generation); and production (local knowledge, availability, seasonality, productivity, intercropping and competition from other crops and processing) criteria. Due to the nature of action research, the criteria were continuously reviewed, taking into account the feedback from the school and kitchen teams during the training workshops; the development of more appropriate techniques for multiplying, harvesting and handling each species; the changes in school menus; and the demand generated by the schools.

We highlight in detail the aspects used in the selection of vegetables, which began with the initial introduction of 210 non-conventional species and varieties, which were subsequently selected for continuous production. This selection was carried out by a multidisciplinary team composed of environmental managers, nutritionists, agronomists and horticulture and biodiversity researchers. Eight issues of particular interest are explained in detail:

1. Existence of nutritional studies or well-established traditional use. The first criterion was to select whether the plants would be considered conventional or not in the schools in the region. This was determined from the list of foods that were sent to schools by the Education Management Unit (UGE). The vegetables sent to these schools were considered conventional. The non-conventional edible plants were identified from lists of non-conventional vegetables (19, 42–47).
2. Good palatability. Palatability was a very important criterion for defining the inclusion of a plant in the project. Even if a vegetable is abundant and easy to grow and prepare, if it does not possess certain sensory characteristics, it will not be consumed. The selection was performed by the project supervisors and based on feedback from the school and kitchen teams, and criteria such as texture, fibrousness, appearance, viscosity, hairiness, bitterness, pungency, acidity, and astringency were considered.
3. Preparation practicality. This criterion was determined over time through feedback from the cooks. Species that were difficult to clean or handle or that made kitchen preparation difficult were excluded from the list because the preparation time for meals at school is limited.
4. Ease of multiplication. This criterion encompasses a large number of processes that were analyzed individually, such as (a) the ability of the plant to produce abundant seeds or propagules; (b) the ability to produce seeds with good viability and germination times of up to 15 days; (c) good viability of the seeds; (d) tolerance to transplanting; (e) the ease of collecting and handling seeds or ease of propagule rooting; and (f) the ease of harvesting the plant or the edible portion. Plants that did not meet any of these criteria were excluded from large-scale production. Modifications to the methodology of multiplication, seedling production, pruning and management methods allowed some species that were previously discarded to be re-established for cultivation.
5. Extended harvest season. The plants that produced most of the year were prioritized over those restricted to specific seasons, with the exception of species growing in cold seasons, when cultivated diversity is lower.
6. Plant cycle. A criterion that was not exclusive but determined a smaller area destined for a species or variety. Plants with a very long cycle, with only one harvest, had a lower priority for cultivation than those with higher yields in the same time interval. Thus, smaller areas were kept for these plants if they met the other criteria.
7. Resistance to pests and adverse weather. Plants that were highly susceptible to pests and diseases relative to others grown in the field were excluded from the list.
8. Post-harvest quality. This was not an exclusion criterion but was relevant in the planning, considering that plants with short or delicate post-harvest periods were produced and shipped on a smaller scale. The minimum shelf life of a vegetable had to be 3 days according to feedback from schools and kitchen teams.

### 2.5 Harvesting and distribution

After the establishment of scale production in 2019, vegetable production started in 2020. Harvests were performed 4x/week and the harvested foods were distributed to 90 local preschools and primary schools, with children aged 0–10 years. Each school received vegetables once a week, and the amount of vegetables received was weighed. During the COVID-19 pandemic in 2020, with the closing of schools, vegetables were sent to families of six schools in deprived neighborhoods, two local hospitals and a charity.

### 2.6 Environmental and nutritional education

To ensure better acceptance and increase knowledge on the delivered vegetables, we worked with school teams, including school gardeners. The goal of this process was to familiarize the children with these vegetables by carrying out observation and cultivation activities, aiming to increase or improve the acceptance of the vegetables in the diet.

The seedlings and seeds of the plants used in the meals were provided to the school gardens. Online and in-person classes lasting a total of 12 h each year on preparing the plant beds, caring for the plants, their multiplication, their uses in teaching subjects, their consumption, and their health benefits were made available to the school teams, and in-person visits were made to monitor the progress of the gardens.

## 3 Results

Based on the above 8 criteria, 34 non-conventional vegetables were selected for cultivation and provided to school kitchens; these vegetables were divided into leafy vegetables, roots, fruits and aromatics Their most common English names, local popular names in Brazil in Portuguese, as well as the parts of the plants used and information on how to prepare them are presented in Tables 1–3.

**Table 1:** Non-conventional leafy vegetables produced on a large scale as part of the *Inova na Horta* project conducted in Jundiaí (São Paulo) from 2020–2023.

Species	Name	Local name in Brazil (cultivated varieties)	Uses	Used parts
*Amaranthus cruentus* L.; *Amaranthus hypochondriacus* L.	amaranth^2^	amaranto (verde/roxa)	sautéed; cooked	leaves and stalks
*Amaranthus viridis* L.	calalu; slender amaranth^2^	caruru	sautéed; cooked	leaves and stalks
*Beta vulgaris* L. var. *esculenta*	yellow beetroot ^2^	beterraba amarela	salad; cooked; baked; juiced with fruits	leaves and stalks, roots
*Brassica carinata* A.Braun i	Abyssinian cabbage^2^	mostarda-africana	salad; sautéed; cooked	leaves and stalks
*Brassica juncea* (L.) Czern.	leaf mustard^2^	mostarda-crioula	salad; sautéed; cooked	leaves and stalks
*Brassica oleracea* L. var. *acephala*	collard green^2^	couve-crioula (verde/roxa/crespa/kale)	salad; sautéed; cooked	leaves and stalks
*Celosia argentea* (L.) Kuntze.	Lagos spinach, common cockscomb^2^	celósia; espinafre-africano	sautéed; cooked	leaves
*Cnidoscolus aconitifolius* (Mill.) I.M.Johnst.	tree spinach; chaya^2^	chaya-mansa	cooked and sautéed; cakes	leaves
*Foeniculum vulgare* Mill.	fennel^2^	funcho	salad; sautéed; cooked; juiced with fruits	leaves; stalks and flower
*Galinsoga parviflora* L.; *Galinsoga quadriradiata* L.	gallant soldier^2^	guasca	sautéed; cooked; seasoning	leaves and stalks
*Glebionis coronaria* (L.) Cass. ex Spach.	chop-suey greens^2^	crisântemo-japonês	salad; sautéed; cooked	leaves and stalks
*Hibiscus acetosella* L.	cranberry hibiscus^2^	vinagreira folha roxa	salad; tea; juice; jam and cakes	leaves
*Ipomoea batatas* (L.) Lam.	sweet potato^2^	batata-doce (roxa larga)	sautéed; cooked	leaves
*Lactuca indica* L.	Indian lettuce^2^	almeirão-de-árvore (branco/roxo)	fresh; sautéed	leaves
*Moringa ovalifolia* Lam.	moringa^2^	moringa	salad; sautéed; cooked	leaves
*Pereskia aculeata* Mill.	Barbados gooseberry^1^; pereskia	ora-pro-nóbis; pereskia	salad; sautéed; cooked; cakes	leaves
*Raphanus sativus* L. *var. oleiferus*	fodder radish^2^	nabo	salad; sautéed; cooked	leaves
*Rumex acetosa* L.	sorrel^2^	azedinha (larga, crespa)	salads, juiced with fruits	leaves and stalks
*Sonchus oleraceus* L.	sow-thistle^2^	serralha	salad; sautéed; cooked	leaves
*Tropaeolum majus* L.	nasturtium^2^	capuchinha	salad; sautéed; cooked	leaves and stalks

The scientific names of more than one species are given when more than one species of similar cultivation and use is used interchangeably. More than one popular name is indicated when no consensus was reached for the popular name. Regarding the popular names in Portuguese, the information in parentheses notes when more than one variety or cultivar within the same species was used throughout the project. The parts sent to schools for consumption are listed, although the plants may have other edible parts.

^1^Data taken from Global Biodiversity Information Facility (GBIF) database available at https://www.gbif.org, accessed 11/12/2022.

^2^Data taken from the Plants For A Future (PFAF) database available at https://pfaf.org/user/Default.aspx, accessed 11/12/2022.

**Table 2:** Non-conventional condiments produced on a large scale as part of the *Inova na Horta* project conducted in Jundiaí (São Paulo) from 2020–2023.

Species	Name	Local name in Brazil (cultivated varieties)	Uses	Used parts
*Allium tuberosum* Rottler ex Spreng.	garlic chives^2^	nirá	salad; sautéed; cooked; seasoning	leaves
*Aloysia citrodora* Palau	lemon verbena^2^	erva-luisa	seasoning; tea; juiced with fruit	leaves
*Curcuma longa* L.	turmeric^2^	cúrcuma	seasoning	rhizome
*Lippia alba* (Mill.) N.E.Br. ex Britton & P.Wilson	bushy lippia^1^	cidreira-de-árvore, melissa	seasoning; tea; juiced with fruit	leaves
*Mentha* ssp.	mint^2^ (peppermint, apple mint, chocolate mint, eau de cologne mint, pineapple mint, spearmint)	mentas (menta, hortelã-maçã, hortelã-chocolate, levante, hortelã-abacaxi, hortelã-verde)	seasoning; tea; salad; juiced with fruit	leaves and stalks
*Ocimum gratissimum* L.	clove basil^1^	manjericão-cravo	seasoning; tea, juiced with fruit; cakes	leaves
*Ocimum* sp.	zaatar basil	manjericão-zaatar	seasoning	leaves

The scientific names of more than one species are indicated when more than one species of similar cultivation and use is used interchangeably. More than one popular name is indicated when no consensus was reached for the popular name. Regarding the popular names in Portuguese, the information in parentheses notes when more than one variety or cultivar within the same species was used throughout the project. The parts sent to schools for consumption are listed, although the plants may have other edible parts.

^1^Data taken from the Global Biodiversity Information Facility (GBIF) database available at https://www.gbif.org, accessed 11/12/2022.

^2^Data taken from the Plants For A Future (PFAF) database available at https://pfaf.org/user/Default.aspx, accessed 11/12/2022.

**Table 3:** Non-conventional tubers, roots, rhizomes, fruits and other plants produced on a large scale as part of the *Inova na Horta* project conducted in Jundiaí (São Paulo) from 2020–2023.

Species	Name	Local names (Brazil)	Uses	Used parts
*Hibiscus sabdariffa* L.	roselle^2^	hibisco, vinagreira	tea; juice; jam and cakes	fruit calyx
*Solanum macrocarpon* L.	African eggplant; gboma	giboma	sautéed; cooked	fruits and leaves
*Colocasia esculenta* (L.) Schott	taro^2^	inhame-roxo	sautéed; cooked	rhizome
*Dioscorea bulbifera* L.	air potato^2^	cará-moela	sautéed; cooked; baked	air tuber
*Helianthus tuberosus* L.	Jerusalem artichoke; sunchoke^2^	tupinambo	cooked; baked	roots
*Ipomoea batatas* (L.) Lam.	sweet potato (purple, orange, yellow, blue, white)^2^	batata-doce (moita, roxa, guarani)	cooked; baked	roots
*Smallanthus sonchifolius* (Poepp.) H. Rob.	yacon^2^	yacon	salad; cooked; baked	roots

The scientific names of more than one species are indicated when more than one species of similar cultivation and use is used interchangeably. More than one popular name is indicated when no consensus was reached for the popular name. Regarding the popular names in Portuguese, the information in parentheses notes when more than one variety or cultivar within the same species was used throughout the project. The parts sent to schools for consumption are listed, although the plants may have other edible parts.

^1^Data taken from the Global Biodiversity Information Facility (GBIF) database available at https://www.gbif.org, accessed 11/12/2022.

^2^Data taken from the database Plants For A Future (PFAF) database available at https://pfaf.org/user/Default.aspx, accessed 11/12/2022.

From January 2020 to June 2023, 12 tons of vegetables were produced and provided to schools, hospitals and charities, of which 9 tons were sent to 90 municipal schools. The quantities produced in 2019 were not computed in this study due to the experimental characteristics of the vegetable cultivation. During the study period, with the reopening of schools after the pandemic and the participation of more schools in the project, production increased but without increasing the cultivated area.

Table 4 shows the amount of each vegetable sent on a weekly basis to the schools from 2020–2023. In 2020, deliveries were sent to families during the pandemic, who received vegetables along with a food kit with information on how to use these vegetables, and to schools after the resumption of classes. The surplus was donated to hospitals, social assistance services and community kitchens in the region, as shown in the Supplementary materials.

**Table 4:** Total amount of non-conventional food plant production sent to municipal schools from 2020–2023 in kilograms.

	Production for school feeding (kilograms)				Total produced (kilograms)
	Families	Schools	Schools	Schools	
	2020	2021	2022	2023*	
Abyssinian cabbage	120		130	17	267
African eggplant fruit			10	74	84
Air potato		90	89	180	359
Amaranth leaves			2	16	18
Barbados gooseberry	160	49	123	170	502
Beetroot leaves		70			70
Bushy lippia		12	106	28	146
Chop-suey greens		10	8	5	23
Clove basil		72	90	10	173
Collard green	240	44	198	9	491
Cranberry hibiscus		24	192	103	319
Fennel			76		76
Fodder radish leaves			50		50
Gallant soldier		55	6	11	72
Garlic chives	186	129	143		458
Indian lettuce	150	122	308	49	629
Jerusalem artichoke		24	76	21	121
Lagos spinach		36			36
Leaf mustard			60		60
Lemon verbena		3	8	22	33
Mint (spearmint, peppermint, apple mint, chocolate mint, eau de cologne mint, pineapple mint)		18	44		62
Moringa leaves		6	11	22	39
Nasturtium	66	9	262	15	352
Roselle	23	56	171	146	396
Slender amaranth			8		8
Sorrel	768	684	912	303	2,667
Sow-thistle		2		23	25
Sweet potato		80		57	137
Sweet potato leaves	155	138	80	59	432
Taro		23		100	123
Tree spinach; chaya		45	140	142	327
Turmeric			7	84	91
Yacon		10	76	218	304
Zaatar basil		15	21	16	52
Total/year	1868	1826	3,406	1901	9,001

**Figure 4:**
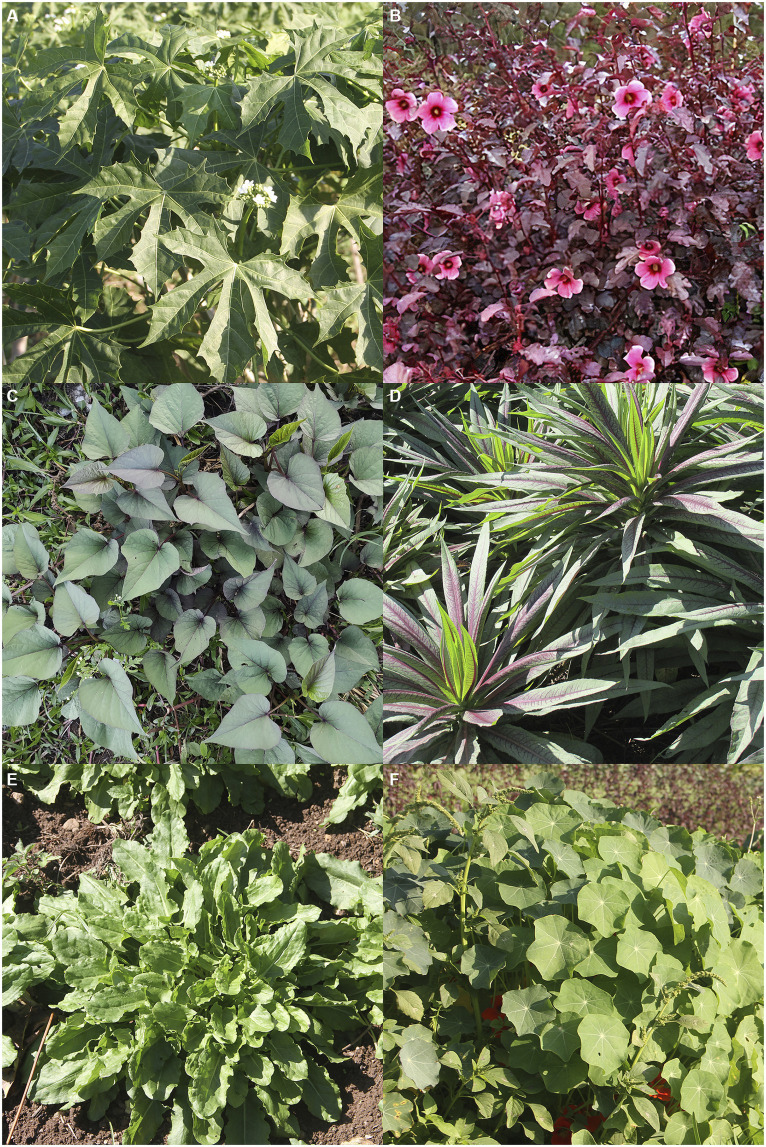
Non-conventional vegetables produced in the largest quantity during the “Inova na Horta” project between 2020 and 2023: **(A)** chaya, *Cnidoscolus aconitifolius*; **(B)** cranberry hibiscus, *Hibiscus acetosella*; **(C)** sweet potato leaves, *Ipomoea batatas*; **(D)** Indian lettuce, *Lactuca indica*; **(E)** sorrel, *Rumex acetosella*; and **(F)** nasturtium, *Tropaeolum majus.*

Leafy vegetables were the largest part of the production (Figure 4), with a total weight of 6,441 kg corresponding to 71.6% of the total harvest. Roots and fruits accounted for 11.3% (1,021 kg), respectively, and herbs and spices accounted for 12.2% (1,091 kg).

Among the leafy vegetables, sorrel (*Rumex acetosella*), sweet potato leaves (*Ipomoea batatas*) and Indian lettuce (*Lactuca indica*) were the most frequently provided. Among the root vegetables, sunchoke (*Helianthus tuberosus*) and air potato (*Dioscorea batatas*) were the two most provided. Garlic chives (*Allium tuberosum*), clove and zaatar basil (*Ocimum* sp.) were produced throughout the year and frequently provided. The roselle calyx (*Hibiscus sabdariffa*), despite not being a real fruit, was considered a fruit by the cooks.

Table 5 lists the nutritional content, based on literature data, of the most produced and consumed vegetables in the project and compares it with the nutritional content of the conventional vegetables. Note the high mineral and protein values of the vegetables produced in the project.

**Table 5:** Nutrient content of selected nutritional food plants and comparison to conventional vegetables.

Name	Status	Local names in Brazil	Protein (g/100 g)		Ca (mg/100 g)		Mg (mg/100 g)		P (mg/100 g)		Fe (mg/100 g)		K (mg/100 g)		Zn (mg/100 g)	
sorrel, leaves	unconventional food plant (PANC)	azedinha*	1.73	[48]	84	[48]	105	[48]	46	[48]	5.87	[49]	623	[48]	0.45	[48]
Indian lettuce, leaves	unconventional food plant (PANC)	almeirão-roxo*	1.32	[49]	154	[49]	0.52	[49]	37	[49]	0.52	[49]	409.41	[49]	0.34	[62]
Nasturtium, leaves	unconventional food plant (PANC)	capuchinha*	5	[48]	73	[48]	0.46	[48]	43	[48]	0.46	[48]	168	[48]	0.76	[48]
Tree spinach, leaves	unconventional food plant (PANC)	chaya*	3.6	[56]	218	[56]	8.17	[56]	155	[56]	8.17	[56]	34	[58]	0.78	[55]
Sweet potato, leaves	unconventional food plant (PANC)	folha de batata-doce*	3.8	[50]	187	[50]	79	[50]	68	[50]	5.43	[50]	375–639	[50]	0.89	[50]
Barbados gooseberry, leaves	unconventional food plant (PANC)	ora-pro-nobis*	2.1	[48]	269	[48]	1.33	[48]	18	[48]	1.33	[48]	323	[48]	0.28	[48]
Cranberry hibiscus, leaves	unconventional food plant (PANC)	vinagreira roxa*	3.87	[52]	464	[52]	144.9	[52]	92	[52]	n.c		340	[52]	n.c	
Lettuce	conventional food plant	alface	1.08	[55]	37.5	[55]	12.7	[55]	26	[55]	0.75	[55]	311	[55]	0.29	[55]
Cabbage	conventional food plant	repolho	1.12	[55]	39.3	[55]	11.3	[55]	16.7	[55]	0.4	[55]	208	[55]	0.17	[55]
Endive	conventional food plant	escarola	1.54	[55]	0	[55]	13.4	[55]	25.1	[55]	0.74	[55]	281	[55]	0.71	[55]
Chicory	conventional food plant	almeirão	1.78	[55]	30.4	[55]	16.7	[55]	46.6	[55]	0.87	[55]	433	[55]	0.34	[55]
Arugula	conventional food plant	rúcula	2.48	[55]	107	[55]	23.9	[55]	31.4	[55]	1.02	[55]	298	[55]	0.31	[55]
Collard greens	conventional food plant	couve	2.87	[55]	208	[55]	42.8	[55]	48.7	[55]	0.66	[55]	557	[55]	0.4	[55]

The asterisk (*) indicates the non-conventional food species grown in the project. The protein and mineral data listed for the vegetables were taken from the literature. All non-conventional vegetables have a higher content of some macro or micronutrient compared to the corresponding conventional vegetable.

### 3.1 Training for teachers and kitchen teams

The schools received training on the use of vegetables for school meals, which included not only kitchen teams but also teachers. The vegetable gardener teams were trained in workshops and subsequently given the non-conventional edible plants for cultivation. For the educators, in-person and remote training sessions were held on eight topics, for a total of 12 h of training, and 8 h of on-call training was available to answer questions. The topics included garden planning, composting, natural disease and pest control, use of non-conventional edible plants, and didactic activities using the garden. The training sessions were attended by all 90 schools involved in the project; however, not all the teachers in these units joined the project. A total of 1,800 professionals participated in the training.

For the kitchen teams, no new recipes were proposed, but suggestions for inclusion within the predetermined daily menu were offered. During the training, the preparation, nutritional aspects, handling, and storage of these vegetables were presented. Kitchen teams from 90 schools were trained in 2.5-h training sessions every year for the duration of the project.

## 4 Discussion

In this study, we reported the feasibility of the production and culinary use of 34 biodiverse, nutrient-rich and underutilized vegetables for school meals in a medium-sized city in Brazil. The selected vegetables had previous nutritional studies available that established their traditional use; good palatability; ease of multiplication; extended harvest season; great resistance to pests and adverse weather; good post-harvest quality; and practicality of preparation.

Over a 3.5-year period, 90 schools received a total of 9 tons of biodiverse vegetables on a weekly basis (56–60). The use of these plants in meals was stimulated by culinary and educational workshops with school cooks and teachers. To our knowledge, this is the first study to show the feasibility of improving the nutritional and biodiverse status of school meals on a large scale.

In Brazil, previous reports describe either one-off limited initiatives carried out in schools, usually on a small scale and not linked to a public policy as is the case of Jundiai. Other studies analyzed the existing public policies and food programs for increasing biodiversity rather than focusing on production (48–52, 61). Our study is the first to show, based on a municipal public policy, the feasibility of selecting and producing 34 PANC to be safely introduced into school meals (62). The advantages of our study include the regular supply of these vegetables, which are not yet commercially available, by centralizing demand and production. In addition, our study contributes to enhancing the availability of biodiverse foods for school meals (63).

The novel aspects of our case study were the introduction of non-conventional vegetables for school meals to the entire municipal network of a medium-sized city; the centralized municipal production and distribution logistics; and training for teachers and kitchen teams, including school gardeners, in a predominantly urban community. The project “*Gulayan sa Paaralan Program* (GPP)” (58) carried out in the Philippines (2007–2014) was an initiative similar to that of the present work. It also had the goal of improving the quality of school food by increasing the biodiversity of meals using local and traditional vegetables. The Philippines project was, however, embedded in a different context, with a school population with a high prevalence of malnutrition, as well as a focus centered on decentralized school gardens. A study carried out after 2 years of the project showed a significant increase in nutritional status and academic performance in 523 children with different degrees of wasting, 123 of whom were severely wasted (62). In our case, the schools were located in an urban region with little space for growing food on a scale that could impact the children’s diet, justifying a more centralized, large-scale production. School gardens had mostly educational purposes, and most educators and cooks were unfamiliar with food production, which seemed to be different from the situation in the Philippines, in which vegetables seemed to be known locally. In the current project, most of the vegetables were not frequently used or known to the local community, which increased the amount of training required for kitchen and educator teams (64).

In Kenya, a project from 2017 promoted the purchase of food for schools from local farmers, including traditional African leafy vegetables. This project provided incentives for local farmers to grow African native vegetables for school purchase, while in the Jundiaí case, production was carried out in a centralized garden. The common thread between the two projects was the focus on the use of leafy, fast-growing vegetables with high nutritional value and on educational and culinary activities using the biodiverse vegetables (63).

### 4.1 Nutrient-rich food plants

Previous bromatological analyses (39, 55) have shown that the vegetables selected in this study are nutrient-rich, with higher amounts of important minerals and proteins than conventional vegetables. However, it is important to note that these vegetables are not yet included in the national food composition database, despite existing data supporting their nutritional value and benefits. For instance, as shown in Table 5, the calcium content of hibiscus cranberry (*Hibiscus sabdariffa*) leaves is 11 times greater than that of a vegetable with a similar use, the common lettuce. Sorrel (*Rumex acetosella*) and cranberry hibiscus contain two or even three times more magnesium than the conventional vegetable with the highest content of this mineral, collard greens (*Brassica oleracea* var. *acephala*). Iron is often deficient in children’s food, and “chaya” or tree spinach (*Cnidoscolus aconitifolius*) contains 12 times more iron than does collard greens, of similar use. Zinc, another fundamental mineral for child development, is present in nasturtium (*Tropaeolum majus*) at concentrations two and a half times greater than those in arugula and four times greater than those in cabbage. Nasturtium also has twice as much protein as the conventional vegetable with the highest protein content and the same uses, arugula (39, 55).

The advantages of using these nutrient-rich and underutilized plants are several: they are easier to propagate and cultivate, they are less affected by pests, and they can be produced in larger quantities and for a longer period than their conventional counterparts (39, 55). The increase in biodiversity in the diet therefore allows the complementation of several nutrients that are present in smaller quantities in conventional vegetables, and the non-conventional vegetables can be considered nutrient sources with low production cost that are simple to use. Additionally, they are suitable for various types of diets, considering that their consumption is associated with a greater content of fiber, vitamins, and other nutraceutical compounds not evaluated in this study (29).

### 4.2 Project development

Although we have shown the feasibility of large-scale vegetable production for biodiverse school meals, some points must be considered. The project involved multiple actors and a large logistic chain, requiring personnel training to ensure that vegetables were properly cultivated and prepared following sanitary requirements (65, 66). Institutional support and funding were therefore essential for project implementation since non-conventional vegetables might have an initial higher implementation cost than conventional vegetables. Our project provided the opportunity for formal training to farmers, cooks and education teams to cultivate, propagate and make culinary use of these plants, broadening their repertoire.

### 4.3 Centralized field production

The importance of traditional or indigenous plants in the human diet has been gaining attention in several continents (64, 65), but less attention has been given to the importance of these plants in school meals, especially in countries with high food insecurity levels. We demonstrated that producing biodiverse school meals with non-conventional plants on a large scale is possible, and we believe that our project can be replicated in different environments. Although our species selection included several plants native to different continents, the selection of species must be adequate for the characteristics of the cultivation area, considering the climate of the region, its seasonality, the type of soil, the irrigation system, and other characteristics that affect the growth and management of these species. We also highlight the importance of determining how to obtain seedlings and propagules that are not produced commercially on a large scale.

For some of the cultivated plants, there were no cultivation systems available, and our study tested different arrangements to ensure production. The intercropping of these plants with conventional vegetables also needs to be better explored since research in this area is still incipient. The inclusion of non-conventional edible plants in areas cultivated with conventional vegetables can maximize production by taking advantage of niches with conditions under which conventional vegetables do not thrive. As ease of reproduction was also a criterion in the selection of species, our methodology allowed the project to be self-sufficient in the multiplication of both perennial and annual species. We also highlight the strong potential of leafy perennials, which remain in the field longer, require less labor and less soil preparation, and reduce production costs, some of which are also less demanding in terms of fertility and management.

In summary, we developed a “farm-to-table” methodology in which 34 biodiverse food plant species (Tables 1–3) were selected for large-scale production for school meals. This methodology could be replicated by municipalities of various sizes as a food and nutritional security public policy aimed at increasing the supply of fresh food with high nutritional value, associated with the valorization of local biodiversity and adaptation to climate change. Decentralized production by a local farmer network or in urban and school gardens is also possible. Because the selected vegetables are resistant, nutritious, and long-cycle species, when produced under agroecological management, they can guarantee nutritional security for children by providing fresh food with high nutritional value. The incidence of stunting in children is expected to increase as a consequence of climate change and the accompanying economic disparities. Improving the knowledge on the cultivation of alternative and more resilient vegetables could help mitigate the worsening of food insecurity in the near future.

As future prospects, we believe that the selection of the 34 PANC based on cultivation, culinary preparation, nutritional value and palatability performed in this study could be used as an initial basis for incorporating more biodiversity in school meals and incrementing national food policies related to biodiversity. There is also a need for further studies on the impact of these interventions on children’s health.

## 5 Conceptual or methodological constraints

Our project has some limitations. We did not obtain specific data on the acceptability of the plants by the children, but school dietitians reported that the acceptance of non-conventional edible plants was similar to that of conventional vegetables. Another important limitation is the lack of analysis of children’s health after the introduction of school meals with PANC. Due to the difficulty in finding propagules, the project required an initial year for species multiplication before being able to establish a constant shipment of vegetables to the schools. The project required skilled labor and training hours, whether in the multiplication and maintenance of germplasm or in field management and harvesting, involving extra costs for the municipality. Studies evaluating the impact on the nutritional aspects of PANC introduction in culinary preparations are still lacking.

## Author contributions

GR: Conceptualization, Data curation, Investigation, Project administration, Writing – original draft, Writing – review & editing. NM: Investigation, Supervision, Writing – review & editing. BS: Supervision, Writing – review & editing. MT: Methodology, Project administration, Writing – review & editing. MD: Data curation, Funding acquisition, Supervision, Writing – review & editing. AB: Funding acquisition, Project administration, Resources, Writing – review & editing. TM: Conceptualization, Supervision, Validation, Writing – original draft, Writing – review & editing.
