# Supplementary material for: Large-scale production of non-conventional edible plants for biodiverse school meals
Source: Front Nutr. 2024 Apr 30;11:1282618. doi: 10.3389/fnut.2024.1282618 (PMC11091910; doi:10.3389/fnut.2024.1282618)
Supplement: Supplementary file 1 [file Table_1.DOCX]

Supplementary Material

# Supplementary Data

**Supplementary Table 1.** Non-conventional vegetable over-produce donated to institutions in Jundiai 2020-2023, in kilograms.

|  | **Over-produce donated (kg)** | | | | | | | | **Total donated** |
| --- | --- | --- | --- | --- | --- | --- | --- | --- | --- |
|  | **Universitário Hospital (hospital)** | | **Mesa Brasil (Institution)** | | | **São Vicente Hospital (hospital)** | | **Centro Popular (Institution)** |  |
|  | **2020** | **2021** | **2020** | **2021** | **2022** | **2020** | **2021** | **2022** |  |
| air potato | 24 |  |  | 24 |  |  |  |  | 48 |
| amaranth leaves |  |  | 8 |  |  | 16 |  |  | 24 |
| barbados gooseberry | 40,5 |  |  |  |  | 36 | 12 |  | 88,5 |
| chop-suey greens | 26 | 10 |  | 10 |  | 26 |  |  | 72 |
| clove basil |  |  |  |  |  | 6 |  |  | 6 |
| collard green | 108 |  |  |  | 12 | 84 |  |  | 204 |
| cranberry hibiscus |  | 45 |  | 45 |  |  |  |  | 90 |
| fodder radish leaves | 37,5 |  |  |  |  | 37,5 |  |  | 75 |
| gallant soldier | 10 |  | 80 |  |  | 16 |  |  | 106 |
| garlic-chives |  | 6 | 30 | 6 |  | 12 | 18 |  | 72 |
| indian lettuce | 137,5 | 10 | 10 | 10 | 25 | 117,5 |  |  | 310 |
| jerusalen artichoke |  | 100 | 52 | 100 |  | 6,5 |  |  | 258,5 |
| lagos spinach | 7,5 |  |  |  |  | 7,5 |  |  | 15 |
| leaf mustard | 66 | 15 |  |  | 64 | 90 | 4 | 32 | 271 |
| leaf mustard |  |  |  |  |  |  |  |  | 0 |
| lemon verbena | 0,5 |  |  |  |  |  |  |  | 0,5 |
| mint | 12,75 | 6 |  | 6 |  | 18 | 13 |  | 55,75 |
| mostarda | 6 |  | 24 | 15 |  |  |  | 16 | 61 |
| nasturtium | 34,5 |  |  |  | 18 | 64,5 | 8 |  | 125 |
| roselle |  | 25 | 10 | 25 |  |  |  |  | 60 |
| sorrel | 153 | 90 | 102 | 84 | 156 | 159 | 54 | 18 | 816 |
| sweet potato leaves | 22,5 |  |  |  | 12 | 22,5 | 8 |  | 65 |
| taro |  |  |  |  | 12 |  |  |  | 12 |
| tree spinach; chaya | 10,5 | 51 | 9 | 51 |  | 13,5 | 15 |  | 150 |
| yacon |  |  | 14 |  |  | 3,5 |  |  | 17,5 |
| zaatar basil | 3 |  |  |  |  |  |  |  | 3 |
|  |  |  |  |  |  |  |  |  |  |
| Total/year | 699,75 | 358 | 339 | 376 | 299 | 736 | 132 | 66 | **3005,75** |

This table refers to the total amount of non-conventional vegetables donated to municipal hospitals and charities, the amount of harvests carried out during school vacation periods.
